# Dose-response relationship between physical activity and anxiety symptoms in medical students from Serbia

**DOI:** 10.3389/fspor.2025.1606002

**Published:** 2025-08-22

**Authors:** Konstantinos Stratakis, Jovana Todorovic, Momcilo Mirkovic, Dejan Nešić, Zorica Terzić-Šupić

**Affiliations:** ^1^ Faculty of Medicine, University of Belgrade, Belgrade, Serbia; ^2^ Faculty of Medicine, Institute of Social Medicine, University of Belgrade, Belgrade, Serbia; ^3^ Faculty of Medicine, University of Pristina-Kosovska Mitrovica, Kosovska Mitrovica, Serbia; ^4^ Faculty of Medicine, Institute for Medical Physiology, University of Belgrade, Belgrade, Serbia

**Keywords:** physical activity, mental health, dose-response, young adults, medical students

## Abstract

**Introduction:**

Despite growing evidence supporting the mental health benefits of physical activity (PA), the nature of its relationship with anxiety symptoms remains uncertain in student populations. This study aimed to investigate differences in mean scores on the Zung Anxiety Scale among groups of students with different levels of PA and to determine whether there is a dose-response relationship between PA and anxiety symptoms, taking into account various sociodemographic and lifestyle factors and study year.

**Methods:**

Cross-sectional data were collected from 1,266 fifth-year medical students at five Serbian universities during two academic years. PA was assessed using the International Physical Activity Questionnaire (IPAQ), and categorized into three MET-min/week groups: <600, 601–6,000, and >6,000. Anxiety symptoms were measured using the Zung Self-Rating Anxiety Scale. Logistic regression was used to estimate odds ratios for anxiety by PA category, accounting for sociodemographic and lifestyle factors.

**Results:**

Students engaging in 601–6,000 MET-min/week of PA were less likely to report anxiety symptoms compared to those engaging in less than 600 MET-min/week. No additional benefit was observed among participants exceeding 6,000 MET-min/week, suggesting a plateau effect. Compared to students in the <600 MET-min/week group, those in the 601–6,000 MET-min/week group were less likely to study in Novi Sad and reported an average or good self-rated financial status, but were more likely to consume alcohol. Participants in the >6,000 MET-min/week group were more likely to be male and belong to the 2023–2024 academic year, and less likely to report an average financial status compared to their peers in the <600 MET-min/week group. Female sex was negatively associated with being in the >6,000 MET-min/week group compared to the 601–6,000 MET-min/week group.

**Conclusion:**

The most significant protective benefits against anxiety symptoms were seen in individuals who participated in PA for 601 to 6,000 MET-minutes per week. No additional benefits were observed for PA levels exceeding 6,000 MET-minutes, suggesting a potential plateau effect. These findings emphasize the importance of promoting sustainable PA habits among students. Future research is needed to establish causality and examine the underlying psychophysiological mechanisms in the relationship between PA and mental health.

## Introduction

Regular physical activity (PA) enhances motor skills and functional capabilities, significantly contributing to overall independence and physical well-being (1). Research indicates that incorporating daily PA and/or exercise into one's lifestyle can reduce the risk of chronic diseases and mortality, serving as a means of primary disease prevention (2). The World Health Organization (WHO) recommends that adults aged 18 to 64 engage in 150 to 300 min of moderate-intensity aerobic PA or, 75 to 150 min of vigorous-intensity PA each week, or an equivalent combination of both (3). Individuals who follow the WHO's PA recommendations have a significantly lower risk of all-cause mortality compared to those who do not (4). Implementing daily PA and exercise interventions can lead to an 80% reduction in the risk of cardiovascular disease (CVD) (5), a 90% reduction in the risk of type 2 diabetes (6), and a 33% reduction in the risk of cancer (7).

In addition to its well-documented benefits for physical health, regular PA is associated with enhanced cognitive functions, such as learning and memory retention, as well as improved mental health (8). Regarding the effect of PA on anxiety, systematic reviews and meta-analyses have shown that regular PA significantly reduces both the severity of anxiety across various populations and age groups. For instance, a meta-analysis by Wipfli et al. (9) found that exercise interventions led to a moderate reduction in anxiety symptoms, comparable to established psychotherapeutic treatments (9). More recently, a comprehensive umbrella review by Singh et al. (10) concluded that PA interventions effectively reduce symptoms of both depression and anxiety, primarily when conducted at moderate intensity (10). These findings provide strong empirical support for incorporating PA into preventive and therapeutic strategies for anxiety disorders.

While the positive effects of PA on mental health are well established, the dose-response relationship—indicating how changes in mental health outcomes are associated with the quantity of PA—has shown nonlinear associations and inconsistent findings. Studies conducted among Canadian adults and adolescents from Europe and North America have shown curvilinear relationships between PA and mental health (11, 12). For instance, Bernard et al. (11) found that moderate levels of objectively measured PA were associated with better self-reported mental health in Canadian adults, while higher levels did not provide additional benefits (11). Similarly, Khan et al. (12) concluded that for adolescents in Europe and North America, PA was positively associated with mental well-being only up to a certain threshold, beyond which the benefits plateaued (12).

Further research from the United States and China has revealed more pronounced nonlinear associations (13, 14). Chekroud et al. (13) identified an inverted U-shaped curve among U.S. adults, suggesting that mental health was optimized at around 45 min of PA, three to five times per week (13). Poorer outcomes were observed with insufficient or excessive levels of exercise, particularly for those engaged in high-intensity or high-frequency training (13). Likewise, Zhou et al. (14) reported a reverse J-shaped relationship in a large Chinese sample, indicating that both low and excessively high levels of PA were linked to worse mental health outcomes (14).

These findings imply that higher levels of PA may lead to either a plateau or a decline in mental health benefits. Both physiological factors, such as overtraining and elevated cortisol levels, as well as psychological factors like maladaptive coping and exercise dependence, have been proposed as explanations for this phenomenon (13). However, the point of diminishing returns regarding the protective effects of PA on anxiety varies across the studies mentioned. Possible explanations for these discrepancies include inconsistencies in measuring mental health outcomes and the neglect of other health behaviors that may also influence mental health. Relying solely on a single-item question about general mental health can introduce randomness in data collection and obscure the associations between PA and common mental health disorders (15). Additionally, various sociodemographic and lifestyle factors have been shown to influence the severity of symptoms of mental health disorders, making it essential to consider these factors when examining the relationship between different levels of PA and mental health (15).

Young adults have historically been at a higher risk for various mental health issues compared to other age groups (16). Students in particular represent a distinct group of young adults who encounter various stressors throughout their educational journeys. These stressors can include moving to a new city, achieving financial independence, forming romantic relationships, and assuming new responsibilities within their communities. All of this occurs while they strive to balance work and personal life within a demanding schedule (17). Adopting healthy lifestyle habits, such as engaging in regular PA, can significantly help these individuals manage stress during this critical stage of life (18). Moreover, young adults who adopt healthy behaviors are more likely to sustain these habits in the future (19).

Emerging evidence indicates that the connection between PA and mental health encompasses both physiological and psychological aspects (20, 21). Hamidi et al. (20) discovered that psychological well-being serves as a mediator between health-promoting behaviors and death anxiety among older adults who have experienced COVID-19 (20). Although this study did not focus specifically on PA or student populations, its proposed mechanism may provide insights into how psychological well-being could mediate the relationship between PA and anxiety among students. Additionally, Dehkordi and Chtourou (21) emphasized that structured PA can positively influence anxiety by improving emotional regulation, increasing self-efficacy, and enhancing coping strategies (21). Together, these findings underscore the importance of considering both physiological and psychological pathways when examining how PA and other health-promoting behaviors can serve as modifiable factors in the prevention and management of anxiety, especially in high-stress environments like universities.

The aim of this study was to investigate differences in mean scores on the Zung Anxiety Scale among groups of students with different levels of PA and to determine whether there is a dose-response relationship between PA and anxiety symptoms, taking into account various sociodemographic and lifestyle factors and study year. Our research aims to provide new insights into whether increasing levels of PA continue to offer protective effects against anxiety symptoms or if they reach a point at which these benefits diminish. Additionally, we seek to contribute to the literature by clarifying the point at which the positive effects of PA on anxiety plateau.

## Methods

### Study design

We analyzed data from two cross-sectional studies conducted during the 2019/2020 and 2023/2024 school years involving fifth-year medical students from five universities in Serbia: Belgrade, Novi Sad, Kragujevac, Niš, and Kosovska Mitrovica. The first study, which took place during the 2019/2020 academic year, included 573 participants, while the second study, conducted during the 2023/2024 academic year, involved 730 participants.

Both studies gathered data from medical students during practical classes in Social Medicine during the winter semester. All students present in the class during the study week received information about the study and were asked to voluntarily complete a paper questionnaire. The response rates were 54.06% for the 2019/2020 study and 68.9% for the 2023/2024 study, resulting in an overall response rate of 61.5%. A total of 1,266 participants completed all the questions in the surveys.

### Research instrument

The instrument used in this study was a questionnaire divided into five sections: (1) socio-demographic and (2) socio-economic characteristics (university, place of residence, sex, age, relationship status, and self-rated financial status); (3) lifestyle and health status characteristics (body weight, height, self-rated health, alcohol use, binge drinking, smoking, cannabis use, and the time spent playing video games and time on social media per day); (4) PA assessed using the International Physical Activity Questionnaire Short Form (IPAQ SF) (22); and (5) anxiety symptoms, measured with the Zung Anxiety Scale (23). Additional details about the study design, participants, and the instruments used can be found in other publications (24, 25).

### Variables

Participants were classified based on their energy expenditure measured in MET minutes per week. This classification was performed according to the World Health Organization (WHO) recommendations for PA (3). The WHO recommends a minimum of 150–300 min of moderate-intensity PA or 75–150 min of vigorous-intensity PA per week, corresponding to an energy expenditure of 600–1,200 MET-minutes per week. We used the upper limit of these recommendations (1,200 MET-minutes per week) as a cutoff value for grouping participants.

A total of 20 variables were analyzed: study year, university, place of residence, sex, age in years, body mass index, relationship status, grade point average, number of meals per day, self-perceived financial status, family relationships, self-related health, use of any tobacco product, alcohol use, binge drinking, cannabis use, time spent playing video games per day in hours, time on social media in hours per day, Zung anxiety scale score, social support, and study engagement score.

### Statistical analyses

The statistical analyses were conducted using descriptive, inferential, and multivariate statistics. Differences between groups for categorical variables were assessed using the Chi-square test, while numerical variables with a normal distribution were analyzed using univariate analysis of variance (ANOVA). For continuous variables without a normal distribution, the Kruskal–Wallis test was employed. The normality of distribution was evaluated using the Kolmogorov–Smirnov test. All variables that were shown significant, were then included in the following multivariate logistic regression models: energy expenditure of 601–6,000 MET-minutes per week compared to 0–600 MET-minutes per week as the outcome variable; energy expenditure of >6,000 MET-minutes per week compared to energy expenditure of 0–600 MET-minutes per week as an outcome variable; and energy expenditure of >6,000 MET-minutes per week compared to energy expenditure of 601–6,000 MET-minutes per week as an outcome variable. Multicollinearity was assessed using the Variance Inflation Factor (VIF), with all predictors yielding VIF values below 5, indicating no significant multicollinearity. Model adequacy was evaluated using the Hosmer–Lemeshow goodness-of-fit test. All analyses were performed using IBM SPSS Statistics, version 22.0.

## Results

The mean and median Zung anxiety scale scores were the lowest in the group with an energy expenditure of 4,801–6,000 MET-minutes per week. There were no significant differences between the scores on the Zung anxiety scale between the group with the lowest energy expenditure and the groups with 6,001–7,200 MET-minutes/week and with >7,200 MET-minutes/week. The mean and median scores for each energy expenditure category are presented in Table 1.

**Table 1:** The mean and median scores on the zung anxiety scales at different energy expenditures.

Expenditure in MET-minutes/week	Zung score Mean ± SD	Zung score Median	*p*-value	*p*-value *post hoc* compared to 0–600
0–600	38.46 ± 9.19	37.00	0.0001	
601–1,200	35.20 ± 7.57	34.00		0.003
1,201–2,400	35.11 ± 8.66	34.00		0.001
2,401–3,600	34.98 ± 8.11	33.00		0.001
3,601–4,800	33.52 ± 8.35	32.00		0.001
4,801–6,000	31.19 ± 7.17	30.00		0.001
6,001–7,200	34.56 ± 10.43	34.00		0.069
>7,200	37.50 ± 9.15	35.00		0.489

After merging the categories that significantly differed from the category with the lowest energy expenditure, and then the categories with higher energy expenditure that showed no differences in score on zung anxiety scale compared to the lowest energy expenditure category, total of 136 students were in the category of 0–600 MET-minutes/week (10.7%), 1,038 were in the 601–6,000 MET-minutes per week (82.0%) and total of 92 students (7.3%) were in the category >6,000 MET-minutes/week.

**Figure 1:**
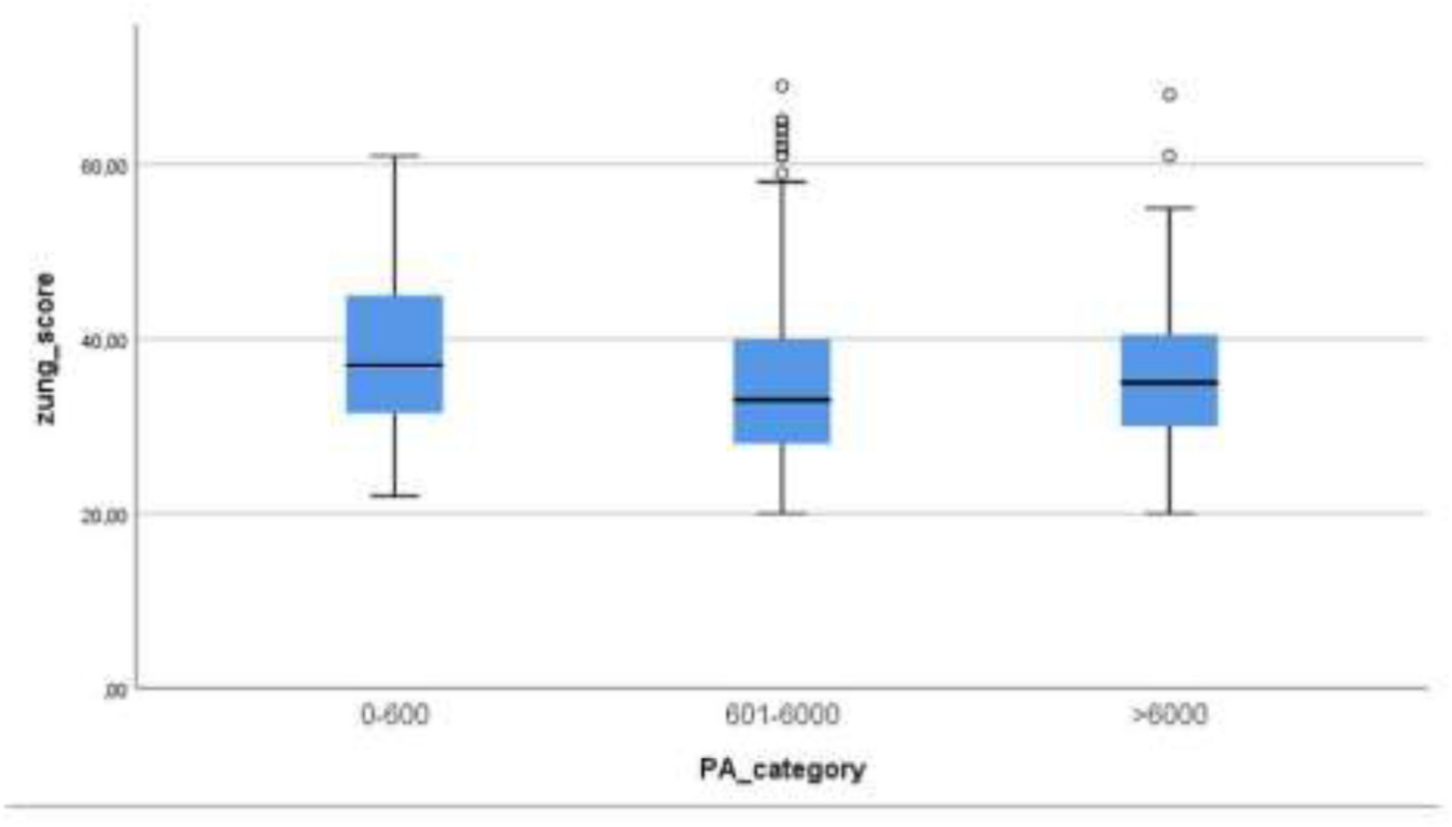
Bar chart with mean scores on zung anxiety scale for the groups based on the energy expenditure.

The mean Zung anxiety scale score for the group with 601–6,000 MET-minutes/week was 34.65 ± 8.24, while the mean Zung anxiety scale score for the group >6,000 MET-minutes/week was 36.61 ± 9.59. The mean scores in the examined groups are presented in Figure 1.

The prevalence of energy expenditure of >6,000 MET-minutes/week was higher in 2023 (8.9% vs.5.1%), among students in Belgrade University (9.3%), among male participants (11.3% vs. 5.0%), among participants with poor self-rated financial status (17.5%), and poor self-rated health (16.2%). The prevalence of energy expenditure of >6,000 MET minutes/week was higher among participants who reported alcohol use (7.4% vs. 6.6%), binge drinking (11.3% vs. 5.3%), and cannabis use (13.3% vs. 6.2%). The participants across different PA categories exhibited significant differences in various socio-demographic, lifestyle, and mental health characteristics, including age, GPA, BMI, time spent playing video games, number of meals consumed, Social Support Scale scores, and Zung Anxiety Scale scores. The characteristics of participants in all three groups are presented in Table 2.

**Table 2:** Differences between the participants.

Characteristics	0–600 MET-minutes/week	601–6,000 MET-minutes/week	>6,000 MET-minutes/week	*p*-value
Study year				
2019/2020	73 (13.3)	446 (81.5)	28 (5.1)	0.002
2023/2024	63 (8.8)	592 (82.3)	64 (8.9)	
University				
Belgrade	66 (9.3)	579 (81.4)	66 (9.3)	0.001
Kosovska Mitrovica	18 (14.2)	102 (80.3)	7 (5.5)	
Nis	3 (7.7)	101 (86.3)	7 (6.0)	
Novi Sad	25 (23.8)	78 (74.3)	2 (1.9)	
Kragujevac	18 (8.7)	178 (86.4)	10 (4.9)	
Place of residence				
Urban	124 (11.2)	907 (81.6)	80 (7.2)	0.468
Rural	11 (8.1)	113 (83.1)	12 (8.8)	
Sex				
Male	40 (9.5)	332 (78.7)	50 (11.8)	0.001
Female	96 (11.5)	697 (83.5)	42 (5.0)	
Age in years X ± SD	24.19 ± 1.99	23.85 ± 1.52	23.55 ± 1.49	0.003
Body mass index in kg/m^2^ X ± SD	22.51 ± 3.29	22.63 ± 3.49	23.54 ± 3.20	0.006
Relationship status				
Single	74 (11.5)	527 (81.7)	44 (6.8)	0.599
In a relationship	62 (10.0)	511 (82.3)	48 (7.7)	
GPA X ± SD	8.63 ± 0.73	8.77 ± 0.74	8.87 ± 0.72	0.027
Number of meals per day X ± SD	2.86 ± 0.95	3.07 ± 0.90	3.47 ± 1.33	0.001
Self-rated financial status				
Poor	8 (14.0)	39 (68.4)	10 (17.5)	0.004
Average	58 (11.0)	444 (84.1)	26 (4.9)	
Good	69 (10.2)	552 (81.5)	56 (8.3)	
Family relationship				
Poor	7 (12.3)	43 (75.4)	7 (12.3)	0.381
Average	25 (13.0)	156 (81.3)	11 (5.7)	
Good	104 (10.3)	835 (82.4)	74 (7.3)	
Self-rated health				
Poor	5 (13.5)	26 (70.3)	6 (16.2)	0.003
Average	32 (16.5)	154 (79.4)	8 (4.1)	
Good	95 (9.4)	843 (83.5)	72 (7.1)	
Use of any tobacco product				
Yes	33 (11.7)	231 (81.6)	19 (6.7)	0.800
No	103 (10.5)	807 (82.1)	73 (7.4)	
Alcohol use				
Yes	104 (9.7)	887 (82.9)	79 (7.4)	0.023
No	32 (16.3)	151 (77.0)	13 (6.6)	
Binge- drinking				
Yes	46 (11.3)	315 (77.4)	46 (11.3)	0.001
No	82 (10.1)	684 (84.5)	43 (5.3)	
Cannabis use				
Yes	17 (8.7)	152 (77.9)	26 (13.3)	0.001
No	119 (11.1)	886 (82.7)	66 (6.2)	
Time spent playing video games per day in hours X ± SD	0.40 ± 1.19	0.31 ± 0.81	0.58 ± 1.14	0.017
Time on social media in hours per day X ± SD	3.09 ± 3.50	2.58 ± 2.17	2.73 ± 2.57	0.744
Zung score X ± SD	38.46 ± 9.19	34.65 ± 8.24	36.61 ± 9.59	0.001
Study engagement X ± SD	33.00 ± 22.44	32.90 ± 8.02	32.64 ± 9.52	0.167
Social support	5.84 ± 1.06	6.14 ± 0.84	6.21 ± 1.01	0.004

Multivariate logistic regression analysis with the energy expenditure of 601–1,200 MET-minutes/week compared to 0–600 MET-minutes/ week showed the association with studying in Novi Sad (OR: 0.32, 95% CI: 0.14–0.76), average (OR: 0.09, 95% CI: 0.01–0.76), or good self-rated financial status (OR: 0.10, 95% CI: 0.01–0.87), alcohol use (OR: 2.79, 95% CI: 1.38–5.65), and score on Zung anxiety scale (OR: 0.96, 95% CI: 0.93–0.99).

Multivariate logistic regression analysis with the energy expenditure of more than 6,000 MET-minutes/week compared to 0–600 MET-minutes/ week showed the association with 2023/2024 study year (OR: 1.69, 95% CI: 1.16–2.45), female sex (OR: 0.13, 95% CI: 0.02–0.72), and average self-rated financial status (OR: 0.04, 95% CI: 0.002–0.68).

Multivariate logistic regression analysis with the energy expenditure of more than 6,000 MET-minutes/week compared to with the energy expenditure of 601–1,200 MET-minutes/week showed the association with female sex (OR: 0.35, 95% CI: 0.14–0.89), and score on Zung anxiety scale (OR: 1.06, 95% CI: 1.01–1.11). The results of the multivariate analyses are presented in Table 3.

**Table 3:** Multivariate logistic regression analyses.

Characteristics	601–6,000 MET-minutes/week vs. 0–600 MET-minutes/week	>6,000 MET-minutes/week vs. 0–600 MET-minutes/week	>6,000 MET-minutes/week vs. 601–6,000 MET-minutes/week
Study year			
2019/2020	1.0	1.0	1.0
2023/2024	1.19 (0.99–1.43)	1.69 (1.16–2.45)	1.17 (0.93–1.47)
University			
Belgrade	1.0	1.0	1.0
Kosovska Mitrovica	0.99 (0.44–2.32)	0.70 (0.12–4.01)	0.49 (0.13–1.89)
Nis	1.09 (0.41–2.88)	0.83 (0.13–5.42)	0.87 (0.27–2.83)
Novi Sad	0.32 (0.14–0.76)	0.00 (0.00–0.00)	0.00 (0.00–0.00)
Kragujevac	1.18 (0.50–2.78)	0.29 (0.05–1.58)	0.63 (0.23–1.74)
Sex			
Male	1.0	1.0	1.0
Female	1.00 (0.50–2.01)	0.13 (0.02–0.72)	0.35 (0.14–0.89)
Age in years	0.90 (0.80–1.03)	0.82 (0.57–1.20)	0.74 (0.52–1.06)
Body mass index in kg/m^2^	0.97 (0.89–1.07)	0.86 (0.69–1.07)	0.99 (0.87–1.13)
GPA	0.95 (0.65–1.39)	1.19 (0.51–2.79)	1.10 (0.65–1.86)
Number of meals per day	1.04 (0.78–1.38)	1.70 (0.97–1.67)	1.28 (0.90–1.81)
Self-rated financial status			
Poor	1.0	1.0	1.0
Average	0.09 (0.01–0.76)	0.04 (0.002–0.68)	0.59 (0.13–2.29)
Good	0.10 (0.01–0.87)	0.07 (0.004–1.26)	0.53 (0.12–2.43)
Self-rated health			
Poor	1.0	1.0	1.0
Average	1.14 (0.27–4.92)	0.59 (0.02–15.15)	0.62 (0.06–6.41)
Good	1.49 (0.38–5.82)	0.80 (0.04–17.72)	0.96 (0.11–8.41)
Alcohol use			
Yes	2.79 (1.38–5.65)	2.62 (0.51–13.51)	0.54 (0.18–1.66)
No	1.0	1.0	1.0
Binge- drinking			
Yes	0.55 (0.29–1.03)	0.35 (0.10–1.26)	1.78 (0.80–3.99)
No	1.0	1.0	1.0
Cannabis use			
Yes	1.02 (0.45–2.33)	0.71 (0.16–3.17)	0.90 (0.33–2.45)
No	1.0	1.0	1.0
Time spent playing video games per day in hours	0.87 (0.68–1.11)	0.97 (0.57–1.67)	1.06 (0.72–1.57)
Zung score	0.96 (0.93–0.99)	1.01 (0.93–1.07)	1.06 (1.01–1.11)
Social support	1.28 (0.96–1.71)	1.89 (0.95–3.79)	1.11 (0.69–1.78)

## Discussion

To our knowledge, this is the first study from Serbia to investigate differences in mean scores on the Zung Anxiety Scale among groups of students with different levels of PA and to determine whether there is a dose-response relationship between PA and anxiety symptoms, taking into account various sociodemographic and lifestyle factors and study year. Among the participants, 10.7% were in the category of 0 to 600 MET-minutes per week of PA, while 82.0% were in the range of 601 to 6,000 MET-minutes per week of PA, and 7.3% exceeded 6,000 MET-minutes per week of PA. Overall, 89.3% of participants met the WHO recommendations for PA. Our findings align with a systematic review that assessed the physical activity and fitness levels of university students, concluding that they generally exhibit satisfactory levels of PA and fitness (26).

The multivariate logistic regression analysis revealed that individuals engaging in 601 to 6,000 MET-minutes of physical activity (PA) per week were 4% less likely to experience anxiety symptoms compared to those in the 0 to 600 MET-minute group, and 6% less likely than those exceeding 6,000 MET-minutes per week. No significant difference in the likelihood of experiencing anxiety symptoms was observed between the group with 0 to 600 MET minutes and the group exceeding 6,000 MET-minutes per week. This pattern illustrates that while PA offers protective effects against anxiety symptoms, these effects tend to diminish after a certain volume of PA.

Regular PA is known to promote the release of neurotransmitters such as endorphins and dopamine, as well as hormones like serotonin, all of which can help reduce anxiety symptoms (27). Furthermore, various psychological mechanisms—such as increased self-efficacy, improved sleep quality, and a lower physiological stress response—may help explain how PA protects against anxiety (28). These mechanisms have been described in models like the stress-buffering model and self-determination theory, which could inform future interventions (29).

Our results indicated that the protective effects of PA against anxiety plateau after 6,000 MET-minutes per week. Several interpretations could explain this plateau in the relationship between PA and mental health. One possibility is that the mental health benefits of PA may reach a saturation point, suggesting that beyond a certain level of PA, additional exercise does not provide significant advantages. Another explanation could be reverse causality; for example, individuals experiencing higher anxiety may engage in increased PA as a coping mechanism, resulting in higher PA levels without a corresponding decrease in anxiety symptoms. To fully understand these phenomena, future longitudinal studies are needed.Our findings align with a large population-based study that used UK Biobank data, which also indicated a similar threshold effect in the relationship between PA and anxiety (30). Additionally, a nationally representative cross-sectional study demonstrated that while moderate levels of PA are beneficial for mental health, engaging in excessive PA does not provide additional benefits (31). Furthermore, another study involving adolescents from North America and Europe confirmed the non-linear relationship between PA and mental health (12).

This body of evidence emphasizes the importance of integrating PA as a modifiable factor in promoting mental health, especially in the post-pandemic era, where concerns about student mental health are increasing. However, it is essential not to recommend excessive PA as part of these mental health strategies.

Regarding study year and university, participants from 2023 study group were more likely to be in >6,000 MET-minutes per week of PA than 0–600 MET-minutes per week of PA and studying in Novi Sad was negatively associated with 601–6,000 MET-minutes per week of PA when compared to less than 600 MET-minutes per week of PA. To our knowledge, there is insufficient evidence to compare our findings on disparities in PA levels between different universities in Serbia. Conversely, the observation that participants from the 2023 group had a higher likelihood of engaging in over 6,000 MET-minutes per week of PA compared to those with 0 to 600 MET MET-minutes per week of PA contradicts evidence suggesting that, in recent decades, physical activity levels among the general population, including young adults, have steadily declined (32).

Female participants were more likely to report engaging in less than 600 MET minutes per week of PA compared to those in either the 601 to 6,000 MET-minutes per week of PA or the over 6,000 MET-minutes per week of PA group. These findings align with a recently published repeated cross-sectional study that analyzed data from the Eurobarometer (2005–2022) involving adults from 28 European countries and examined gender differences in meeting PA guidelines. The authors concluded that, overall, men tend to be more physically active than women (33).

The multivariate logistic regression analysis indicated that participants engaging in 601 to 6,000 MET-minutes per week of PA were more likely to report an average or good self-rated financial status compared to those who participated in fewer than 600 MET minutes per week of PA. Similarly, participants who exceeded 6,000 MET-minutes per week of PA were also more likely to report an average self-rated financial status than those in the group with less than 600 MET-minutes per week of PA. Overall, our findings suggest a negative association between PA and self-rated financial status. The relationship between financial status and PA has been extensively studied with mixed results. Some evidence suggests that individuals from lower-income families and those living in under-resourced communities tend to be less physically active (34). Conversely, other research shows that individuals with poorer self-reported financial statuses may engage in equal or even higher levels of PA compared to their wealthier peers, possibly due to the availability of cost-free activities such as unstructured play and outdoor recreation (35).

In comparison to the group with less than 600 MET-minutes per week of PA, those engaging in 601–6,000 MET-minutes per week of PA showed a positive association with alcohol use. Our findings regarding the relationship between alcohol consumption and PA are consistent with evidence indicating that regular PA often coincides with certain risk behaviors (36). One possible explanation for this trend is that students who are more physically active may form social connections and participate in events where alcohol is present, making them more prone to occasional alcohol consumption (36). Additionally, some individuals may engage in PA as a means of counterbalancing the health risks associated with unhealthy habits, influenced by compensatory beliefs and established patterns (36).

This study has several strengths worth noting. First, it uses a large, multi-center sample of medical students from five different universities, which enhances the regional relevance of the findings. Second, it utilizes valid and reliable research instruments for data collection.

Third, the study employs structured MET-based PA thresholds, similar to those used in other studies (30), allowing for a meaningful interpretation of the results. Finally, the analysis was adjusted for various socio-demographic and lifestyle factors to control for potential confounding variables. These features offer valuable insights for designing future interventions targeting young adults in high-stress academic environments. However, there are several limitations to consider. The cross-sectional design limits our ability to infer causation, and we cannot establish the directionality of the relationship between PA and anxiety. Both PA and anxiety symptoms were self-reported, which may introduce recall bias. Although we adjusted for several confounding factors, we could not include variables such as academic pressure, personality traits, or clinical mental health diagnoses, all of which may influence both PA and anxiety. Lastly, the overall response rates indicate the number of respondents compared to the total number of students enrolled in Social Medicine classes each school year. Students absent during the questionnaire distribution week were considered non-respondents, which may have led to an underestimation of the response rates in both study years.

## Conclusion

To our knowledge, this is the first study from Serbia to investigate differences in mean scores on the Zung Anxiety Scale among groups of students with different levels of PA and to determine whether there is a dose-response relationship between PA and anxiety symptoms, taking into account various sociodemographic and lifestyle factors and study year. The most significant mental health benefits were observed at a weekly PA level of 601 to 6,000 MET-minutes. No additional mental health advantages were found for PA levels exceeding 6,000 MET-minutes, suggesting that a plateau effect may be present. Various sociodemographic and lifestyle characteristics were associated with different PA levels, including study year, university, sex, Self-rated financial status, and alcohol use. Targeted interventions that promote sustainable PA habits within university settings could be beneficial for enhancing student mental health. Future longitudinal and experimental studies are needed to establish causality and examine the underlying psychological mechanisms in the relationship between PA and mental health.

## Data Availability

The original contributions presented in the study are included in the article/Supplementary Material, further inquiries can be directed to the corresponding author.

## References

[1] Miri S Farhadi B Takasi P Ghorbani Vajargah P Karkhah S. Physical independence and related factors among older adults: a systematic review and meta-analysis. Ann Med Surg. (2024) 86(6):3400–8. 10.1097/MS9.0000000000002100 PMC11152881 38846859

[2] Lear SA Hu W Rangarajan S Gasevic D Leong D Iqbal R The effect of physical activity on mortality and cardiovascular disease in 130,000 people from 17 high-, middle-, and low-income countries: the PURE study. Lancet. (2017) 390(10113):2643–54. 10.1016/S0140-6736(17)31634-3 28943267

[3] World Health Organization (WHO). Physical activity. Available online at: https://www.who.int/news-room/fact-sheets/detail/physical-activity (Accessed March 16, 2022).

[4] Geidl W Schlesinger S Mino E Miranda L Pfeifer K. Dose–response relationship between physical activity and mortality in adults with noncommunicable diseases: a systematic review and meta-analysis of prospective observational studies. Int J Behav Nutr Phys Act. (2020) 17:109. 10.1186/s12966-020-01007-5 32843054 PMC7448980

[5] Ades PA Balady GJ Berra K. Transforming exercise-based cardiac rehabilitation programs into secondary prevention centers: a national imperative. J Cardiopulm Rehabil. (2001) 21(5):263–72. 10.1097/00008483-200109000-00003 11591040

[6] Hamman RF Wing RR Edelstein SL Lachin JM Bray GA Delahanty L Effect of weight loss with lifestyle intervention on risk of diabetes. Diabetes Care. (2006) 29(9):2102–7. 10.2337/dc06-0560 16936160 PMC1762038

[7] Warburton DE Nicol CW Bredin SS. Health benefits of physical activity: the evidence. CMAJ. (2006) 174(6):801–9. 10.1503/cmaj.051351 16534088 PMC1402378

[8] Mandolesi L Polverino A Montuori S Foti F Ferraioli G Sorrentino P Effects of physical exercise on cognitive functioning and wellbeing: biological and psychological benefits. Front Psychol. (2018) 9:509. 10.3389/fpsyg.2018.00509 29755380 PMC5934999

[9] Wipfli BM Rethorst CD Landers DM. The anxiolytic effects of exercise: a meta-analysis of randomized trials and dose-response analysis. J Sport Exerc Psychol. (2008) 30(4):392–410. 10.1123/jsep.30.4.392 18723899

[10] Singh B Olds T Curtis R Dumuid D Virgara R Watson A Effectiveness of physical activity interventions for improving depression, anxiety and distress: an overview of systematic reviews. Br J Sports Med. (2023) 57:1203–9. 10.1136/bjsports-2022-106195 36796860 PMC10579187

[11] Bernard P Doré I Romain A-J Hains-Monfette G Kingsbury C Sabiston C. Dose response association of objective physical activity with mental health in a representative national sample of adults: a cross-sectional study. PLoS One. (2018) 13(10):e0204682. 10.1371/journal.pone.0204682 30356252 PMC6200189

[12] Khan A Lee E-Y Rosenbaum S Khan SR Tremblay MS. Dose-dependent and joint associations between screen time, physical activity, and mental wellbeing in adolescents: an international observational study. Lancet Child Adolesc Health. (2021) 5(10):729–38. 10.1016/S2352-4642(21)00200-5 34384543

[13] Chekroud SR Gueorguieva R Zheutlin AB Paulus M Krumholz HM Krystal JH Association between physical exercise and mental health in 1.2 million individuals in the USA between 2011 and 2015: a cross-sectional study. Lancet Psychiatry. (2018) 5(9):739–46. 10.1016/S2215-0366(18)30227-X 30099000

[14] Zhou H Jiang F Liu H Wu Y Tang Y-l. Dose-dependent association between physical activity and mental health, and mitigation effects on risk behaviors. iScience. (2025) 28(2):111866. 10.1016/j.isci.2025.111866 39991549 PMC11847119

[15] Cooney G. Exercise and mental health: a complex and challenging relationship. Lancet Psychiatry. (2018) 5(9):692–3. 10.1016/S2215-0366(18)30291-8 30099001

[16] Pedersen BK Saltin B. Exercise as medicine: evidence for prescribing exercise as therapy in 26 different chronic diseases. Scand J Med Sci Sports. (2015) 25(3):1–72. 10.1111/sms.12581 26606383

[17] Worsley JD Harrison P Corcoran R. Bridging the gap: exploring the unique transition from home, school or college into university. Front Public Health. (2021) 9:634285. 10.3389/fpubh.2021.634285 33816421 PMC8009977

[18] van Sluijs EMF Ekelund U Crochemore-Silva I Guthold R Ha A Lubans D Physical activity behaviors in adolescence: current evidence and opportunities for intervention. Lancet. (2021) 398(10298):429–42. 10.1016/S0140-6736(21)01259-9 34302767 PMC7612669

[19] Lavie CJ Ozemek C Carbone S Katzmarzyk PT Blair SN. Sedentary behavior, exercise, and cardiovascular health. Circ Res. (2019) 124(5):799–815. 10.1161/CIRCRESAHA.118.312669 30817262

[20] Hamidi R Ghodsi P Taghiloo S. The mediating role of psychological well-being in explaining the effect of a health-promoting lifestyle on death anxiety in seniors with COVID-19 experience. Health Nexus. (2024) 2(1):89–98. 10.61838/kman.hn.2.1.10

[21] Dehkordi AM Chtourou H. Managing athlete anxiety: a comprehensive review of psychological interventions in sports psychology. Health Nexus. (2023) 1(4):48–53. 10.61838/kman.hn.1.4.6

[22] Hagströmer M Oja P Sjöström M. The international physical activity questionnaire (IPAQ): a study of concurrent and construct validity. Public Health Nutr. (2006) 9(6):755–62. 10.1079/PHN2005898 16925881

[23] Zung WW. A rating instrument for anxiety disorders. Psychosomatics. (1971) 12:371–9. 10.1016/S0033-3182(71)71479-0 5172928

[24] Stratakis K Todorovic J Mirkovic M Nešić D Tesanovic T Terzić-Šupić Z. Examination of factors associated with physical activity among medical students pre and post-COVID-19 in Serbia. Sci Rep. (2025) 15(1):5791. 10.1038/s41598-025-90544-9 39962148 PMC11832933

[25] Stratakis K Terzić-Šupić Z Todorović J Nešić D Novaković I. Physical activity and mental health of medical students. Cent Eur J Public Health. (2024) 32(1):39–44. 10.21101/cejph.a8097 38669156

[26] Kljajević V Stanković M Đorđević D Trkulja-Petković D Jovanović R Plazibat K Physical activity and physical fitness among university students: a systematic review. Int J Environ Res Public Health. (2021) 19(1):158. 10.3390/ijerph19010158 35010418 PMC8750240

[27] Warburton DER Bredin SSD. Health benefits of physical activity: a systematic review of current systematic reviews. Curr Opin Cardiol. (2017) 32(5):541–56. 10.1097/HCO.0000000000000437 28708630

[28] Anderson ES Wojcik JR Winett RA Williams DM. Social-cognitive determinants of physical activity: the influence of social support, self-efficacy, outcome expectations, and self-regulation among participants in a church-based health promotion study. Health Psychol. (2006) 25(4):510–20. 10.1037/0278-6133.25.4.510 16846326

[29] Gerber M Pühse U. Do exercise and fitness protect against stress-induced health complaints? A review of the literature. Scand J Public Health. (2009) 37(8):801–19. 10.1177/1403494809350522 19828772

[30] Ho FK Petermann-Rocha F Parra-Soto S Boonpor J Gill JMR Gray SR Device-measured physical activity and incident affective disorders. BMC Med. (2022) 20(1):290. 10.1186/s12916-022-02484-0 36064521 PMC9446787

[31] Xu P Huang Y Hou Q Cheng J Ren Z Ye R Relationship between physical activity and mental health in a national representative cross-section study: its variations according to obesity and comorbidity. J Affect Disord. (2022) 308:484–93. 10.1016/j.jad.2022.04.037 35439463

[32] Guthold R Stevens GA Riley LM Bull FC. Worldwide trends in insufficient physical activity from 2001 to 2016: a pooled analysis of 358 population-based surveys with 1.9 million participants. Lancet Glob Health. (2018) 6(10):e1077–86. 10.1016/S2214-109X(18)30357-7 30193830

[33] Owen KB Corbett L Ding D Eime R Bauman A. Gender differences in physical activity and sport participation in adults across 28 European countries between 2005 and 2022. Ann Epidemiol. (2025) 101:52–7. 10.1016/j.annepidem.2024.12.011 39710014

[34] Tandon PS Kroshus E Olsen K Garrett K Qu P McCleery J. Socioeconomic inequities in youth participation in physical activity and sports. Int J Environ Res Public Health. (2021) 18(13):6946. 10.3390/ijerph18136946 34209544 PMC8297079

[35] Eime RM Charity MJ Harvey JT Payne WR. Participation in sport and physical activity: associations with socio-economic status and geographical remoteness. BMC Public Health. (2015) 15:434. 10.1186/s12889-015-1796-0 25928848 PMC4423100

[36] Leasure JL Neighbors C Henderson CE Young CM. Exercise and alcohol consumption: what we know, what we need to know, and why it is important. Front Psychiatry. (2015) 6:156. 10.3389/fpsyt.2015.00156 26578988 PMC4629692

